# Shoot apical meristem and initial vascular development of a late Palaeozoic spermatophyte (order Medullosales)

**DOI:** 10.1093/aob/mcaf336

**Published:** 2026-02-10

**Authors:** Lydéric Portailler, Ludwig Luthardt

**Affiliations:** Institute of Biology, Humboldt-Universität zu Berlin, Unter den Linden 6, Berlin 10117, Germany; Museum für Naturkunde, Leibniz Institute for Evolution and Biodiversity Science, Invalidenstraße 43, Berlin 10115, Germany; Museum für Naturkunde, Leibniz Institute for Evolution and Biodiversity Science, Invalidenstraße 43, Berlin 10115, Germany

**Keywords:** Palaeobotany, early Permian, Pteridospermopsida, Cycadales, seed plant evolution, plant anatomy, apical meristem, evo-devo

## Abstract

**Background and Aims:**

The medullosans are ancient spermatophytes (order Medullosales) with an unusual stem anatomy and have been studied extensively from the late Palaeozoic of North America and Europe. Among the extensive fossil material of *Medullosa*, a single apex has been discovered from the early Permian Chemnitz Fossil Forest. By setting it in an updated functional and developmental context, we aim to provide new knowledge and understanding on the evolution of the vascular development in ancient seed plants.

**Methods:**

We re-examine historical sections of a stem apex of *Medullosa stellata* Cotta, 1832 *emend.* Luthardt *et al.*, 2021 with state-of-the-art stereomicroscopic analysis techniques and digital drawings.

**Key Results:**

The apex exhibits the following unusual combination of features in comparison to modern spermatophytes: (1) an uncommon type of shoot apical meristem with a zone of multiple cell initials only; (2) various coiled leaf primordia completely covering the apex; (3) a flat morphology of the apex indicating considerable primary stem thickening; (4) a primary vascular system of mainly tangentially and horizontally oriented vascular strands; (5) secondary tissues developing centripetally and centrifugally around the primary vascular tissues; and (6) numerous spherical mucilage cavities distributed throughout the sections.

**Conclusions:**

Medullosans exhibited a peculiar stem development not comparable to that of most extant seed plants. Only extant cycads show several specific functional analogies (e.g. a ‘stem-girdling’ vasculature) that might refer to arborescent growth by a pachycaulous stem. This configuration requires sophisticated hydraulic support at an early developmental stage, including vertical and horizontal transportation of water. Our study demonstrates that the stelar evolutionary path of the medullosans was complex and characterized by various adaptive modifications and transitional stages.

## INTRODUCTION

Apical meristematic growth is the primary process of initial cell division and ongoing cellular proliferation, which forms various tissues and, finally, initiates plant-organ development. Therefore, this process determines the overall appearance of a plant, encompassing its architectural, anatomical and morphological features, in addition to their ontogeny (Nishihama and Naramoto, 2020). From an evolutionary perspective, stem apical meristematic activity via cell initials facilitates indeterminate growth in plant organisms and is regarded as a significant innovation of the land plants (Ligrone *et al.*, 2012), although the Charales also have apical meristems (Graham *et al.*, 2000; Kean-Galeno *et al.*, 2024). In a macroevolutionary context, the shoot apical meristem (SAM) evolved into various morphological types in different plant clades, exhibiting numerous innovations across land plants (Kenrick and Crane, 1997; Friedman *et al.*, 2004). The simplest form of SAM occurs in gametophyte and sporophyte shoots of charophyceans and bryophytes, consisting of a single initial cell that divides in a one-, two- or three-dimensional pattern, the last of which enables three-dimensional growth and branching of the shoot (Nishihama and Naramoto, 2020). It was demonstrated in some rhyniophytes of the Early Devonian that the SAM of these tracheophytes consisted of densely packed cell initials, enabling the growth of small, anatomically simple and dichotomizing shoots (Edwards, 1994). In extant tracheophytes, three SAM types are found: the monoplex, simplex and duplex types (Newman, 1965). Some lycopsids and monilophytes possess a monoplex SAM, with a single initial tetrahedral or wedge-shaped cell and a cytohistological zonation into a central, a peripheral and a rib zone (e.g. Stevenson, 1976; Bierhorst, 1977; Cruz and Hetherington, 2025). The simplex-type SAM is characterized by multiple meristematic cells, which differentiate into an outer cytological layer, later forming the epidermal tissues, and an inner layer forming the ground and vascular tissues. The simplex-type meristem occurs predominantly in gymnosperms but also in some lycopsids and monilophytes, whereas the duplex-type SAM is unique to angiosperms (Newman, 1965). In general, it might be suggested that an increasing size and complexity of the SAM, incorporating whole domains of cell initials, was suggestively a macroevolutionary key factor in realizing a more complex architecture and anatomy for spermatophytes and some monilophytes, resulting in increased potential for adaptation to various environmental conditions (Graham *et al.*, 2000; Nishihama and Naramoto, 2020).

In addition to the anatomical and cytohistological features of the cell initials, the SAM also varies in its three-dimensional shape and the position of leaf primordia (e.g. Gola and Banasiak, 2016). In most plants, the apex is cone-shaped, with variable steepness of the flanks. In exceptional cases, when the flanks of the SAM are sub-horizontal, the tip of the SAM can even be a small depression of concave morphology. These different shapes can be found within a single species; for example, the long and short shoots of conifers, where the long shoots have conical apices and the short shoots have dome-shaped apices (Gifford and Wetmore, 1957). Especially in the shoots of Arecaceae and Cycadales, a flat or dome-shaped apex forms from a primary thickening meristem (e.g. Stevenson, 1980; DeMason, 1983).

To investigate plant developmental processes in an evolutionary context, the apical meristem is the most promising region in a plant for studying its ontogenetic growth processes and the origin of anatomical units (Hetherington, 2024). To date, the evolution of apical growth in plants is widely reconstructed from extant plant lineages, because data from the fossil record are exceptionally limited owing to a preservation bias of delicate apical tissue structures, especially in large arborescent plants. There are only a few examples in the literature where fossil shoot or root apical meristems are described in cellular detail (e.g. Delevoryas, 1956; Good, 1971; Good and Taylor, 1972; Ehret and Phillips, 1977; Hetherington *et al.*, 2016; Cruz and Hetherington, 2025). This knowledge gap leaves various open research questions regarding ancestral states and the evolution of primary growth processes, especially in the framework of evo-devo concepts (Friedman *et al.*, 2004; Fouracre and Harrison, 2022; Hetherington, 2024). Fossilized apical meristems of late Palaeozoic tracheophytes, including lycophytes, ferns and calamitaleans, generally show levels of similarity to those of extant relatives (Hetherington, 2024). The fossil record of apical meristems for the seed plant lineage is exceptionally sparse (e.g. Delevoryas, 1956; Good and Taylor, 1972), resulting in a significant lack of information on the evolution of apical meristematic ontogeny and growth processes of this exceptionally diverse group (Fiz-Palacios *et al.*, 2011). To gain a better understanding of the early evolution of spermatophytes, the extinct seed plant groups, such as the Pteridospermopsida, could be of crucial importance (e.g. Doyle, 2006). Among them, the Lyginopteridales and Medullosales, as late Palaeozoic representatives of wet tropical-forest floras during the Carboniferous and early Permian, exhibit an unusual stelar anatomy in comparison to those of other extant and extinct seed plants and are incompletely understood regarding their ontogeny (e.g. Dunn, 2006; Galtier and Meyer-Berthaud, 2006; Luthardt *et al.*, 2021). Medullosan stelar anatomy is unique in having leaf traces produced by several vascular bundles or by the vascular segment surrounding the central zone of parenchyma.

The Medullosales represent a clade of early seed plants (Pteridospermopsida), which were predominantly colonizing late Palaeozoic forest ecosystems of the Northern Hemisphere. Early forms of medullosan are recorded from extensive coal-forming tropical forests of Euramerican lowland basins, spanning from the Mississippian to the late Pennsylvanian (DiMichele *et al.*, 2006; Luthardt *et al.*, 2021). These early medullosans exhibit considerable taxonomic diversity and a variety of growth habits, ranging from climbing, scrambling or leaning to being self-supporting (Wnuk and Pfefferkorn, 1984; Krings *et al.*, 2003). Beyond the palaeotropical lowlands, medullosans were also colonizing seasonally dry forests of the latest Pennsylvanian to early Permian sub-montane basins in central Europe (Luthardt *et al.*, 2021). In contrast to the tropical forms of the Mississippian–Pennsylvanian, these medullosans exhibit a more complex and diverse vascular anatomy, characterized by high abundances of secondary xylem, distinctly taller stems and an arborescent growth habit with a monopodial architecture (e.g. Luthardt *et al.*, 2022).

The early Permian stem taxa were first described by Cotta (1832), who defined *Medullosa stellata* and *Medullosa porosa*. Later, Göppert and Stenzel (1881) described *Medullosa leuckartii*, and Schenk (1889)*Medullosa solmsii*. The latest and most comprehensive study on anatomical characteristics of all these taxa was provided by Weber and Sterzel (1896). Today, the stem anatomy of the medullosans remains puzzling and lacks comparative equivalents in the extant flora, because of the mode of production of leaf traces and the tangential horizontal metaxylem cells (e.g. Rudolph, 1922). Owing to the variable organization of tissues even among individuals, several anatomical varieties have been described for the existing taxa, but a clear differentiation between taxonomically and ontogenetically induced variations is still missing (Göppert and Stenzel, 1881; Weber and Sterzel, 1896). At least some of the varieties exhibit conspicuous characteristics that cannot be attributed to simple growth variations, such as the opposite direction of dominant secondary growth in *M. stellata* var. *typica* and *M. stellata* var. *lignosa* (Luthardt *et al.*, 2021). Understanding the ontogenetic development of the stem vasculature in *Medullosa* will contribute significantly to a more robust taxonomy based only on stem anatomy and provide new insights into the developmental complexity of its unusual vascular organization.

Here, we describe historical sections from the late 19th century, prepared from an anatomically preserved stem apex of an early Permian medullosan seed fern. The specimen is referred to the type species *Medullosa stellata*Cotta, 1832*emend.*Luthardt *et al.*, 2021; it was figured by Weber and Sterzel (1896) but was never described in detail. The original locality of the specimen, the Chemnitz Fossil Forest, represents an early Permian wet forest ecosystem, in which arborescent medullosans were preserved in various states (Rößler *et al.*, 2012; Luthardt *et al.*, 2021, 2022; Rößler, 2021). The apex described here provides unique insights into the morphological and anatomical details of the medullosan apical meristematic zone and the ontogenetic development of its unusual stem-anatomical features.

## MATERIALS AND METHODS

### Geological setting and taphonomic context

**Fig. 1.**
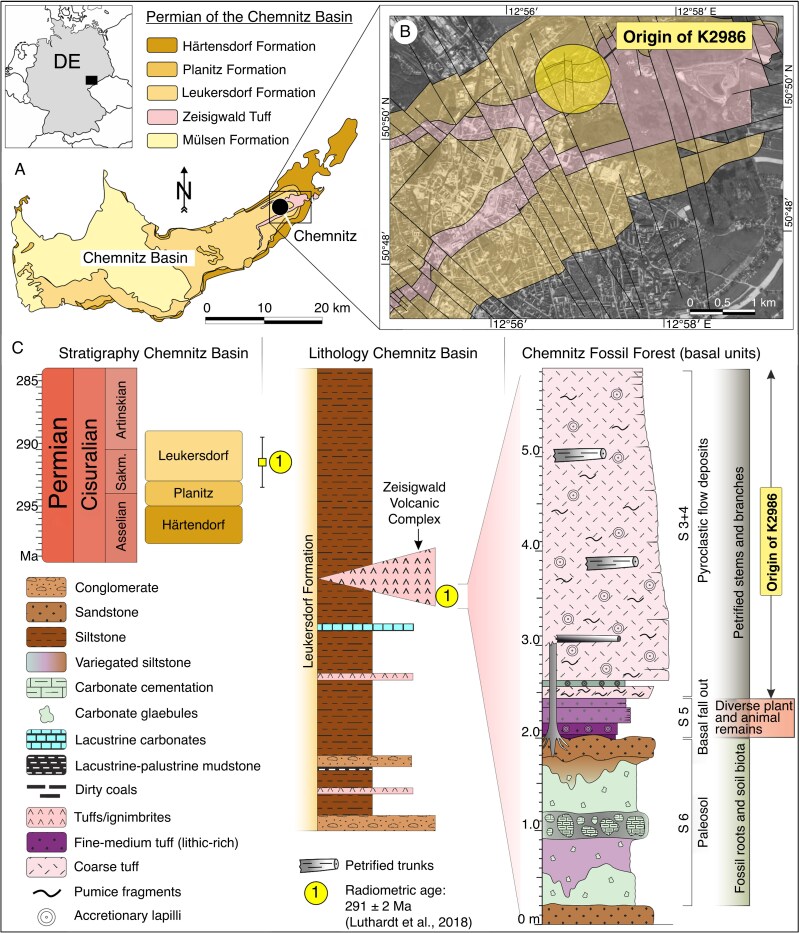
Origin of specimen K2986. (A) Geographical overview on the position and the geology of the Chemnitz Basin. (B) Area of origin of K2986 referred to the outcrop area of the Zeisigwald Pyroclastics in Chemnitz Hilbersdorf (modified from Luthardt *et al.*, 2018). (C) Chrono- and lithostratigraphic framework of the Zeisigwald Pyroclastics and representative lithological profile of the Chemnitz Fossil Forest in Chemnitz Hilbersdorf, including the pyroclastic layer from which K2986 is most likely to originate.

The petrified medullosan fossils on which our study is based originate from the Chemnitz Fossil Lagerstätte, an *in situ* preserved forest ecosystem of early Permian age (Fig. 1), located in central-eastern Germany (Saxony). The Lagerstätte is part of the late Palaeozoic submontane Chemnitz Basin, which was filled predominantly with red clastic sediments of lower to upper Permian age (Schneider *et al.*, 2012). The forested landscape was buried by pyroclastics of the Zeisigwald Volcanic Complex, which is intercalated in the upper Leukersdorf Formation and reveals a radiometric age of 291 ± 2 Mya (Sakmarian–Artinskian; Luthardt *et al.*, 2018). The rapid burial by pyroclastic fallout and flow deposits favoured the *in situ* and partly three-dimensional preservation of (sub-)autochthonous floral and faunal elements, which were all part of the same habitat (e.g. Rößler *et al.*, 2012; Luthardt *et al.*, 2016; Rößler, 2021). The habitat is characterized as a basin-central, seasonally dry forest thriving in sub-humid conditions, benefitting locally from a near-surface groundwater level that might have buffered seasonal fluctuations in the overall moisture regime (Luthardt *et al.*, 2016, 2017). Most of the medullosans were found at the Hilbersdorf locality, where they were forming a taxonomically diverse population in a densely vegetated habitat, together with psaroniaceous tree ferns in the subcanopy to understorey, overtopped by arborescent cordaitaleans, calamitaleans and, probably, Walchian conifers in the canopy (Rößler *et al.*, 2012; Luthardt *et al.*, 2021, 2022; Rößler, 2021). The specimens were found in the basal pyroclastic fallout and overlying pyroclastic density current deposits (Luthardt *et al.*, 2018). Some of them were preserved *in situ* and standing upright at their places of growth, whereas others were broken, transported over short distances and embedded horizontally in the pyroclastic density current deposits.

The anatomically preserved stem segments of the Chemnitz Fossil Lagerstätte were completely petrified by rapid silicification after having been buried by the Zeisigwald pyroclastics. From these acidic ejecta, abundant SiO_2_ and other mineralizing agents, such as fluorite, were released to impregnate the wood and later replace its organic compounds (e.g. Luthardt *et al.*, 2018; Trümper *et al.*, 2018). As a result, wood anatomical features were generally preserved at the cellular level. However, delicate structures, such as parenchymatous cells with non-lignified or poorly lignified walls, were not preserved and were widely replaced by amorphous SiO_2_.

### Studied material

**Fig. 2.**
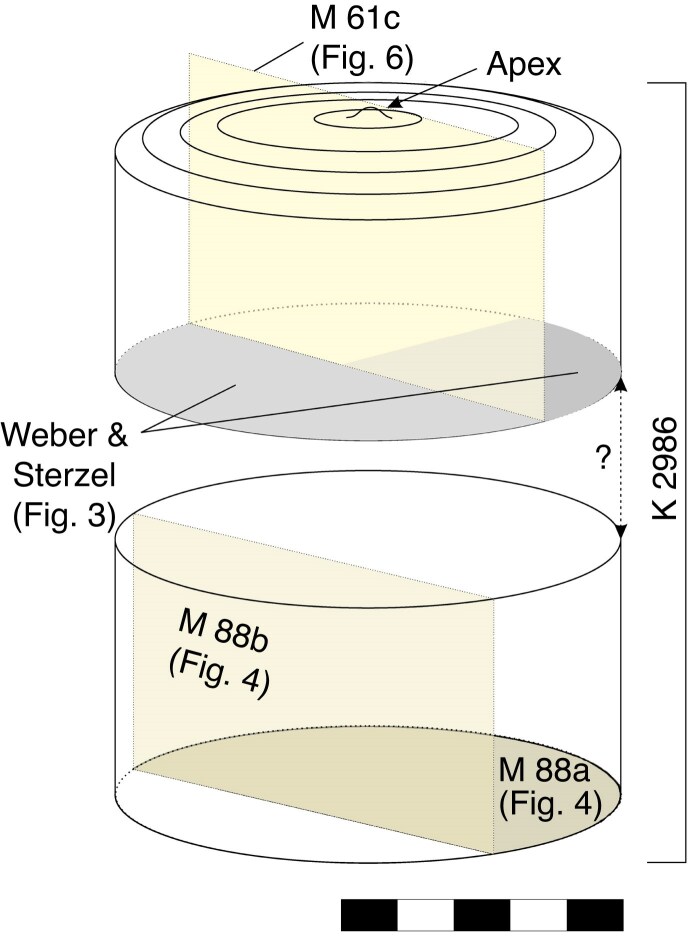
Overview of the various (thin) sections presented in this study and their position in the apical stem.

**Fig. 3.**
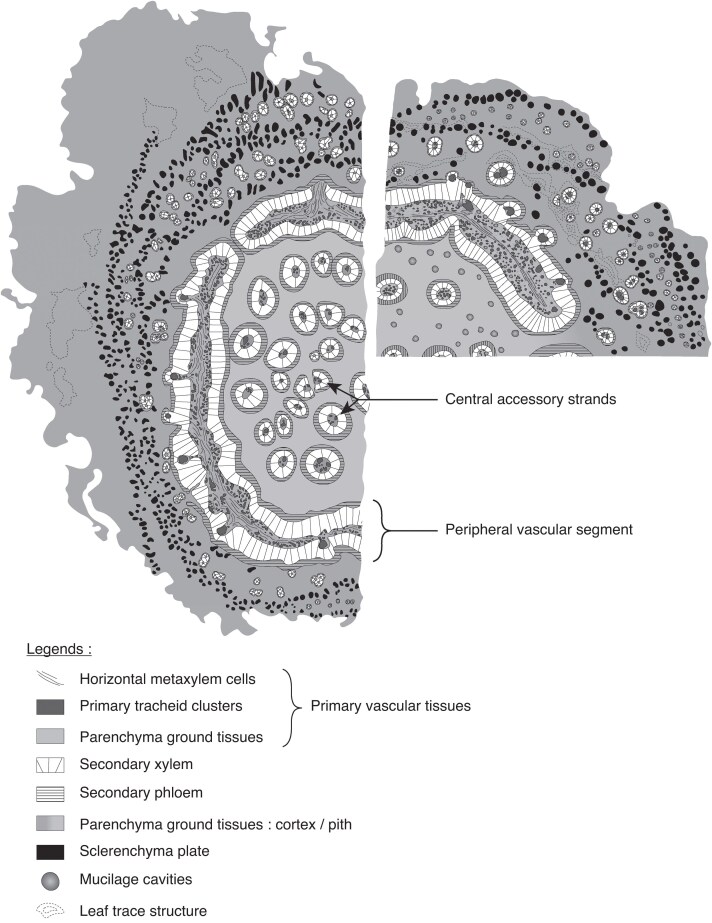
Graphical reproduction of the historical drawings provided by Weber and Sterzel (1896: left, Pl. 1, fig. 2; and right, fig. 7, p. 57), providing the only information on the lost apical stem part, from which M 61c was made. Approximate scale bar: 5 cm.

The apical section of a *Medullosa stellata*Cotta, 1832*emend.*Luthardt *et al.*, 2021 var. *typica*Weber & Sterzel, 1896 stem is studied from various historical sections in transverse and longitudinal orientations (Fig. 2) that were produced in the late 19th century by Otto Weber and later figured by Weber and Sterzel (1896: p. 60, text-fig. 8b). The stem exhibits an exceptional cellular preservation in comparison to most petrified fossils from the Chemnitz Lagerstätte. The study focuses mainly on thin section M 61c, which represents a radial section that was cut nearly through the stem centre, based on Weber and Sterzel (1896: fig. 2, Pl. I and fig. 8b). Two transversal thin sections of the same specimen (M 61 a + b), in addition to the original specimen (collection no. M I) from which the thin sections were prepared, are figured by Weber and Sterzel (1896), but could not be found in any collection. Thus, for detailed information on M 61 a + b, we refer to the historical drawings of Weber and Sterzel (1896), which depict the anatomical features of the cross-section of the specimen (Fig. 3).

**Fig. 4.**
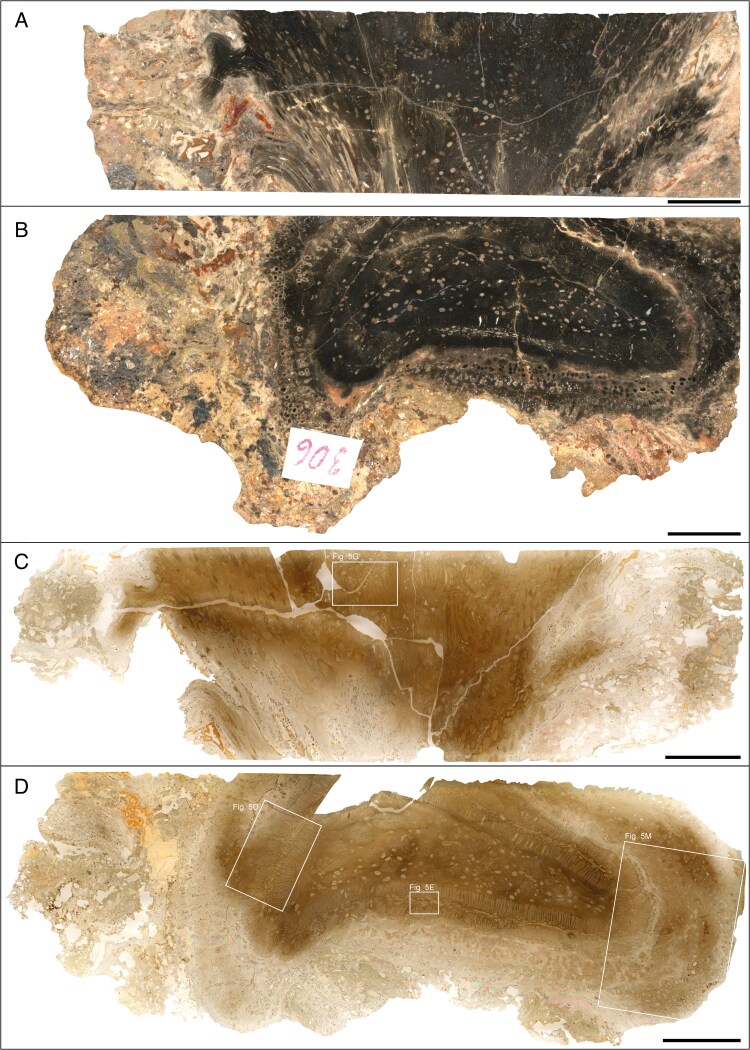
Overview of the different sections of K2986 that are considered to be a more basal section of the same stem as M 61c. (A, C) Longitudinal section passing through the peripheral vascular segment and the cortex (see Fig. 2); C is the thin section M 88b. (B, D) Cross-section of K2986; D is the thin section M 88a. Scale bars: 10 mm.

Additionally, we describe the stem section of K2986 [Fig. 4: longitudinal (Fig. 4A, C) and transverse (Fig. 4B, D) sections], which is interpreted to represent the most proximal segment of the same stem as the apex described above, a concept already proposed by Weber and Sterzel (1896) and Rudolph (1922). The interpretation is here reasonably confirmed, based on: (1) the similar exceptional preservation quality; and (2) widely similar anatomical characteristics that reflect a more mature stage than M 61c. The only difference mentioned by Rudolph (1922) is the shape of the peripheral vascular segment (PVS) in cross-section, which is less circular in K2986. However, variations in the shape of the vascular system might easily result from slightly irregular growth or compressive forces during fossilization. From K2986, three historical thin sections exist that had the original collection number number 138, used by Weber and Sterzel (1896 : p. 62), but were later renamed as M 88a–c. M 88a represents a cross-section, M 88b a tangential section, and M 88c the longitudinal section of a basal petiole attachment that is not described further here.

Today, all the described specimens are stored in the palaeontological collection of the Museum für Naturkunde Chemnitz. The descriptive terminology of the specific anatomical units in *M. stellata* are based on the revised terminology introduced by Luthardt *et al.* (2021).

### Imaging and analysis techniques

All specimens were digitized with an Epson Perfection V600 Photo scanner at resolutions of 2400, 3200 and 4800 dpi. The scans were used as a basis for schematic sketches of the anatomy. In addition, all specimens were analysed microscopically using a Nikon SMZ18 stereomicroscope with ×1.0 and ×1.6 PlanApo objectives. For microscopic photography, we used a Nikon Di6 (12 MP) camera attached to the stereomicroscope and controlled by Nikon Elements-D imaging software (©2024 Nikon Corporation). Most microscopic photographs were produced by automatic image stacking to create the best possible focus. The software was also used to measure cellular dimensions. For the digital production of figures and drawings, we used the Adobe Illustrator 2025 v.29.8.2 (Adobe^®^ Inc., 2025) and CorelDRAW X7 software v.26.0 (CorelDRAW^®^, 2025).

## RESULTS

*Systematics*

*Class Pteridospermopsida*

*Order Medullosales*

*Family Medullosaceae*

*Genus Medullosa* Cotta, 1832 *emend.* Luthardt *et al.*, 2021

*Type species Medullosa stellata* Cotta, 1832 *emend.* Luthardt *et al.*, 2021

*Synonymy. Medullosa stellata* Cotta, 1832 *emend.* Luthardt *et al.*, 2021 var. *typica*Weber & Sterzel, 1896.

*Medullosa stellata* Cotta, 1832 *emend.* Luthardt *et al.*, 2021 var. *corticata*Weber & Sterzel, 1896.

## Description

**Fig. 5.**
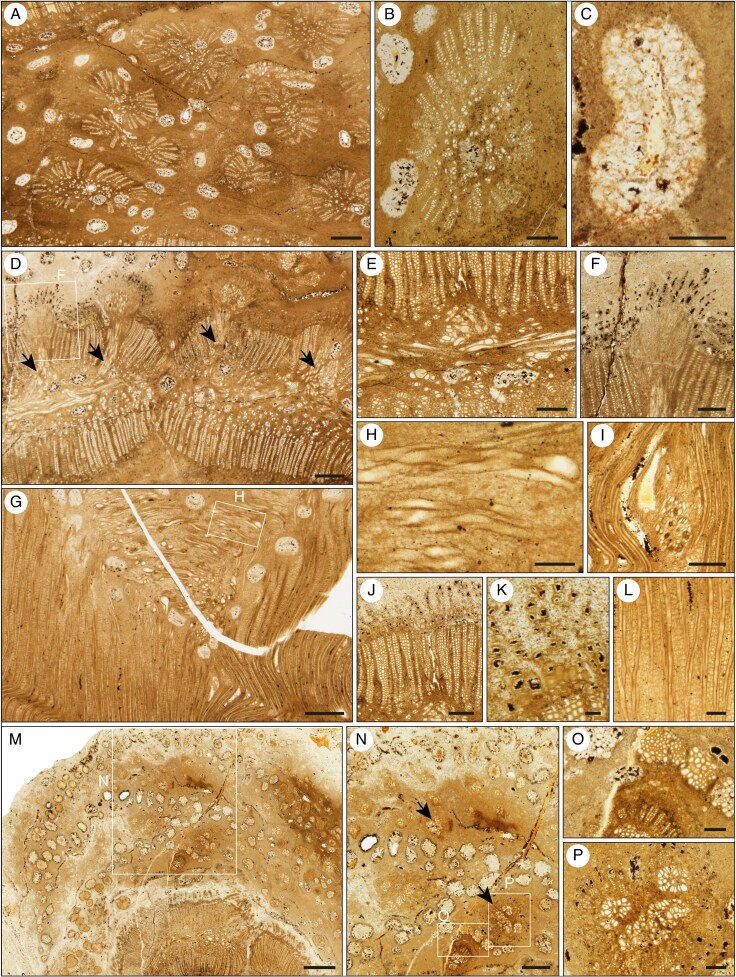
Stem anatomical details of the basal part of K2986 from cross-section M 88a and longitudinal section M 88b (only G–I, L). On every picture of this figure and the following ones (unless otherwise figured), standard orientation is respected. The top of the picture is oriented towards the exterior or towards the top of the specimen, in transverse and longitudinal sections, respectively. (A) Overview of the pith with central accessory strands (CAS) and numerous spherical mucilage cavities. Scale bar: 1 mm. (B) Detail of a CAS with central primary xylem and surrounding secondary xylem. Scale bar: 0.5 mm. (C) Detail of a mucilage cavity with marginally crushed parenchyma cells. Scale bar: 250 µm. (D) Part of the peripheral vascular segment (PVS) with a gap in the vasculature in attachment position of a frond and various leaf traces (arrows) emerging from the central zone of primary xylem. Scale bar: 1 mm. (E) Detail of a very initial leaf trace formed from horizontal metaxylem clusters. Scale bar: 0.5 mm. (F) Detail of a leaf trace emerging from the PVS and entering the cortex. Scale bar: 0.5 mm. (G) Longitudinal section showing secondary xylem and the central zone of primary vasculature in tangential view, together with numerous mucilage cavities. Scale bar: 1 mm. (H) Detail of metaxylem with scalariform wall structure. Scale bar: 250 µm. (I) Emerging leaf trace in the secondary xylem in tangential view. Scale bar: 0.5 mm. (J) Secondary xylem and phloem of the PVS in cross-section. Scale bar: 0.5 mm. (K) Detail of the phloem zone with subangular scelerenchyma cells and crushed phloem cells in between. Scale bar: 100 µm. (L) Loose manoxylic wood of the PVS in tangential view. Scale bar: 250 µm. (M) Distinctly thickened cortex at the position of an attached frond with bands of sclerenchyma strands. Scale bar: 2 mm. (N) Detail of sclerenchyma strands and petiole vascular bundles (arrows). Scale bar: 1 mm. (O) Detail of sclerenchyma cells in the strands (close-up of lower arrow from N). Scale bar: 250 µm. (P) Detail of a petiolar bundle that is dividing into three separate bundles (close-up of upper arrow from N). Note the secondary xylem and phloem surrounding the bundle. Scale bar: 250 µm.

*Near-apical stem section (K2986):* The historical thin sections of K2986 reveal detailed three-dimensional information on the (near-)apical stem anatomy of *M. stellata* var. *corticata* at different ontogenetic levels. The sections of M 88a (transverse) and M 88b (tangential) are positioned a few centimetres below the apex and show the typical anatomical organization of a *M. stellata* (var. *corticata*) stem (Figs 4 and 5), including: (1) a wide parenchymatous pith, in which numerous central accessory strands (CAS) are located; (2) a ring-shaped PVS, with primary and secondary vascular tissues; and (3) a thick parenchymatous cortical zone with leaf primordia (Fig. 4B, D). The whole cross-section of M 88a is 92 mm long and 34 mm wide (Fig. 4D). The elongated pith is 43 mm long and 11 mm wide and composed of isodiametric parenchyma cells and numerous spherical mucilage cavities (Fig. 5A). The pith comprises 21 CAS, representing cauline bundles that are composed of metaxylem and parenchyma in the centre (Fig. 5B). The primary tissues are surrounded by secondary xylem/phloem wedges separated by rays, both resulting from the activity of a bifacial cambium. The metaxylem tracheids measure 40–80 µm in diameter and occur isolated or grouped in clusters of two to four cells. The peripheral clusters are closely associated to the secondary xylem tracheid files, with a smooth transition in growth direction. The tracheid files are two cells wide, on average, and ∼400 µm long. Tracheid diameters measure 40–60 µm. The secondary phloem is poorly preserved and shows remains of a few sclerenchymatic cells. The PVS surrounding the central parenchyma zone extends over a radial diameter of 54 mm. In cross-section, it is best described as a sporadically interrupted ‘ring’, with a central zone of primary vascular tissues and centripetally and centrifugally arranged zones of secondary tissues (Fig. 5D). The total thickness of the PVS is 5.5 mm, on average, with ∼1.3-mm-thick primary vascular zone, and ∼1.4-mm-thick secondary xylem on both sides. In M88 a and b, both zones of secondary xylem are of equal thickness, but the outer secondary phloem is thicker than the inner one.

The primary vasculature of the PVS is composed of numerous metaxylem strands in a tangential and horizontal orientation and positioned in between the centripetal and centrifugal layers of secondary tissue. The horizontal strands are accompanied by vertical metaxylem clusters positioned adjacent to the inner boundary of the centrifugal secondary tissues (Fig. 5D, E). Tracheids measure 55–85 µm in diameter and possess scalariform to multiseriate araucarian pitting (Fig. 5H). Sporadically, metaxylem strands leave the PVS horizontally to form acropetally oriented leaf traces (Fig. 5D–F, I).

The secondary xylem and phloem wedges are intersected and separated by wide multiseriate rays of radially elongated parenchyma cells (Fig. 5G, J–L). Tracheid rows are two to three, sometimes four tracheids wide. Tracheids measure 40–60 µm in diameter and possess walls with multiseriate araucarian pitting on both the radial and tangential walls. The pits have diameters of 7 µm on average.

Individual leaf traces emerge from the radially and horizontally oriented primary vasculature of the PVS and, while still being connected to the PVS, possess their own bifacial cambium when traversing the cortex (Fig. 5O, P). The leaf traces have the same tissue composition as the PVS and CAS and expand through the cortical zone as leaf vascular bundles. These vascular bundles frequently divide into two to three smaller bundles, each with an average diameter of 11.6 mm (Fig. 5O, P). The vascular bundles are grouped in peripherally elongated areas several millimetres in length that are separated from each other by numerous sclerenchyma strands (Fig. 5M, N). The sclerenchyma strands are ∼450 µm in diameter and composed of thick-walled sclerenchyma cells (Fig. 5O). Leaf traces are then composed of a vascular bundle and a sclerenchyma bundle that can anastomose, and several leaf traces enter a single frond. The cortex itself is 9 mm thick, on average, but distinctly thicker at the attachment position of a frond petiole, and is composed of parenchymatous ground tissue (Fig. 5M). Mucilage cavities are rare in the cortex, measuring ∼300 µm in diameter.

**Fig. 6.**
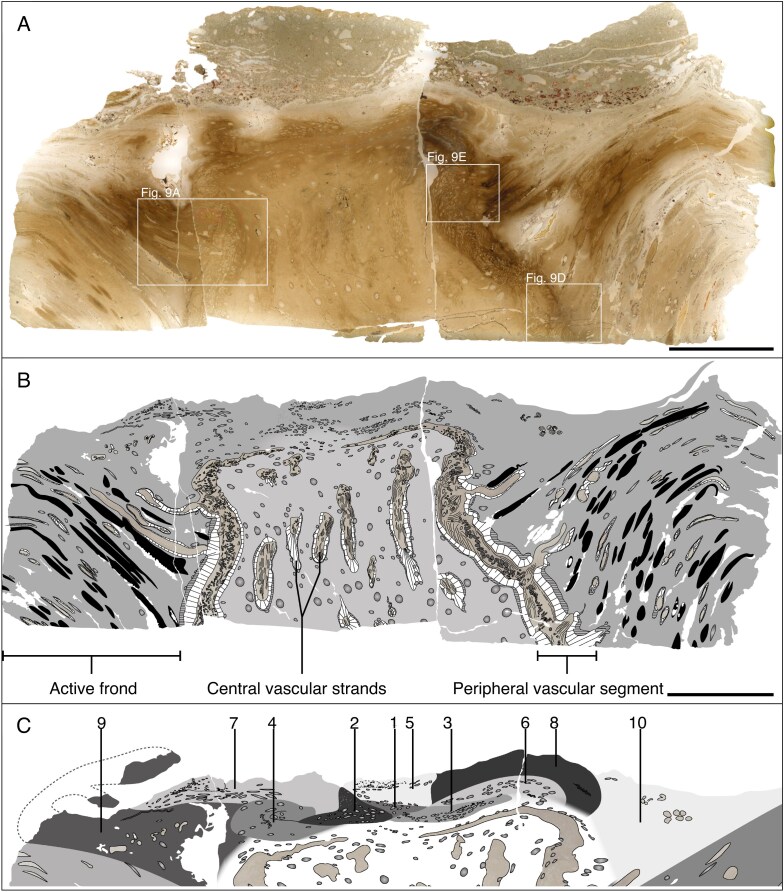
Stem-apical radial thin section of M 61c. (A) Overview scan. (B) Descriptive drawing with all tissues represented. Surrounding sediments and non-connected tissues are not represented. For keys, see Fig. 3. (C) Interpretative drawing of the hypothetical position of leaf primordia (LP) and the two active fronds, with primary vascular tissues and mucilage cavities represented. The numbers are pointing to the different LP in hypothetical ontogenetic order. The dashed line in LP 9 is a hypothetical reconstruction of the axes, based on the position of the remaining tissues, showing the beginning of the frond dichotomization process. Scale bars: 10 mm.

**Fig. 7.**
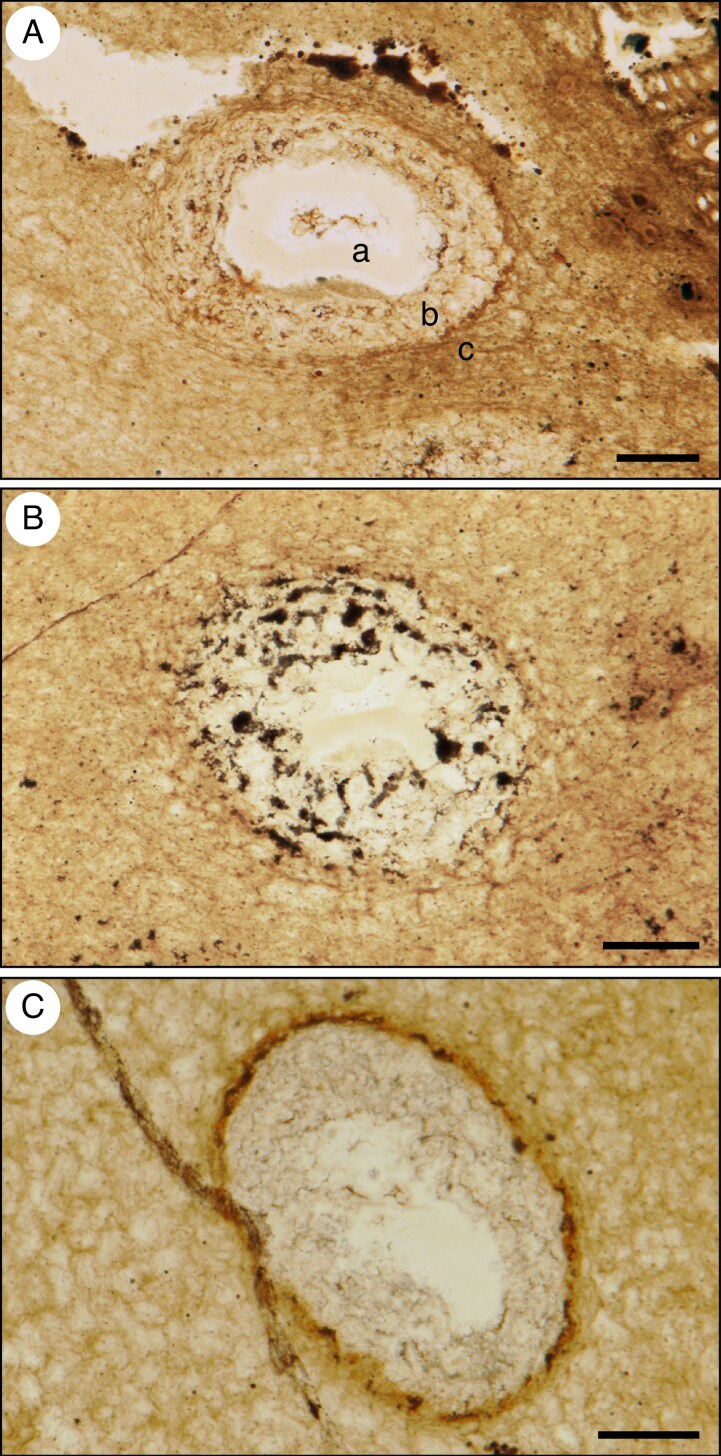
Details of mucilage cavities from the central zone of parenchyma in M 61c. (A) Spherical structure with empty cavity (a), a marginal zone of altered parenchyma cells (b) and deformed surrounding parenchyma tissue attributable to volume extension of the cavity (c). (B) Mucilage cavity with blackish remains of unknown compounds. (C) Mucilage cavity with only slightly deformed marginal parenchyma cells. Scale bars: 200 µm.

In general, the frequent occurrence of mucilage cavities (Figs 5A and 6) is among the most conspicuous features of the stem-apical part of *M. stellata* (var. *corticata*). The cavities are present in all tissue regions of the stem, including the pith, vascular tissues, cortex, leaf primordia and basal frond petioles. Morphologically, the cavities are described as globular to elliptical hollow spaces (Fig. 5C and 7). In the pith, they are spherical in shape, ranging from 300 to 1000 µm in diameter. Everywhere else, they are predominantly elongate and extend from 100 to 1600 µm in long-axis diameter. Cellular details reveal a hollow space where organic remains of potential ergastic substances are preserved as undefined dark contents (Fig. 7B). The peripheral cells around the cavities represent modified, thin-walled parenchyma cells that are partly crushed or tangentially elongated (Fig. 7).

**Fig. 8.**
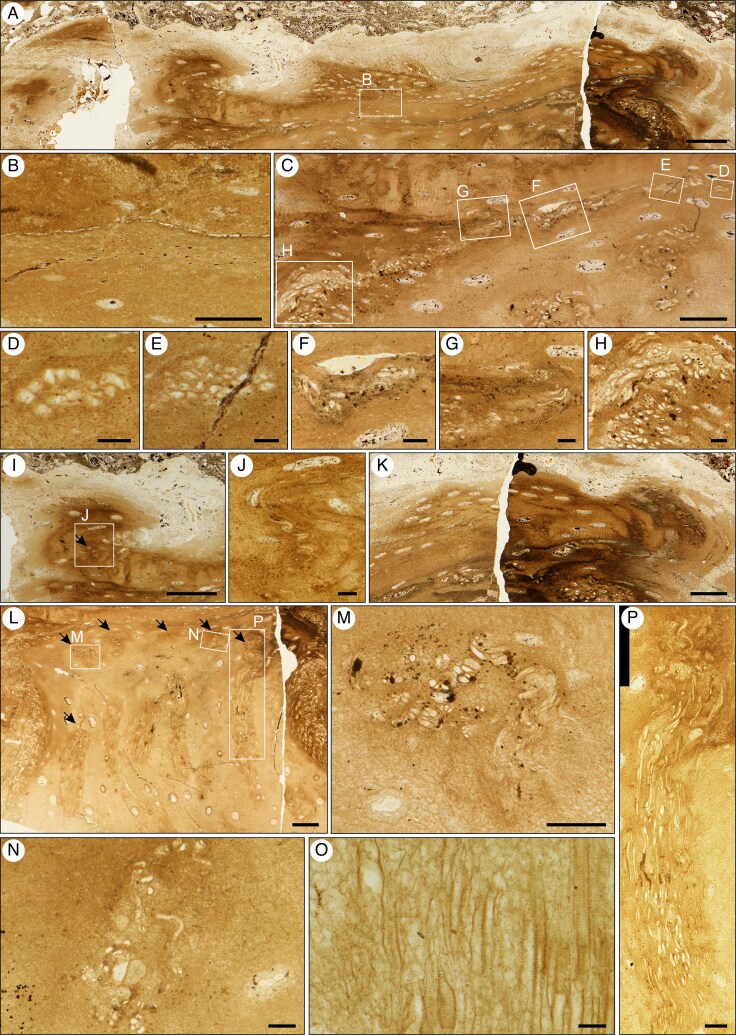
Microscopic details of the stem-apical radial section of M 61c. (A) Microscopic panorama of the apex with overtopping leaf primordia. Scale bar: 2 mm. (B) Detail of the apical meristem with dark-filled cellular structures and a tissue demarcation against the overlying leaf primordia (for interpretation, see also Fig. 10). Scale bar: 0.5 mm. (C) Overview of the initial peripheral vascular segment (PVS) with primary bundles of horizontally oriented tracheids. Scale bar: 1 mm. (D–H) Row of ontogenetic development of the primary vasculature, starting with horizontal procambial strands (seen in cross-section; D, E), later being replaced by horizontal metaxylem (F–H). Note the connection of the primary girdling vasculature with a leaf primordium (G) and the initiation of the secondary vasculature with (sub-)vertical metaxylem tracheids in H. Scale bars: 100 µm in D, E; 200 µm in F–H. (I) Leaf primordium with a coiled habit, showing initial vasculature (arrow) and mucilage cavities. Scale bar: 2 mm. (J) Detail of meandering provascular elements in a leaf primordium. Scale bar: 250 µm. (K) Another coiled leaf primordium on the right side of the section. Scale bar: 1 mm. (L) Overview of the pith with various central accessory strands (CAS) initiating directly below the initial vasculature (arrows). Scale bar: 2 mm. (M) Detail of an initial CAS with chaotically arranged metaxylem tracheids. Scale bar: 0.5 mm. (N) Same details as in M, for another initial CAS. Scale bar: 200 µm. (O) Metaxylem tracheids of a CAS with scalariform pitting. Scale bar: 100 µm. (P) Microscopic panorama of a CAS, showing the development of primary and secondary conducting tissues. Scale bar: 0.5 mm.

**Fig. 9.**
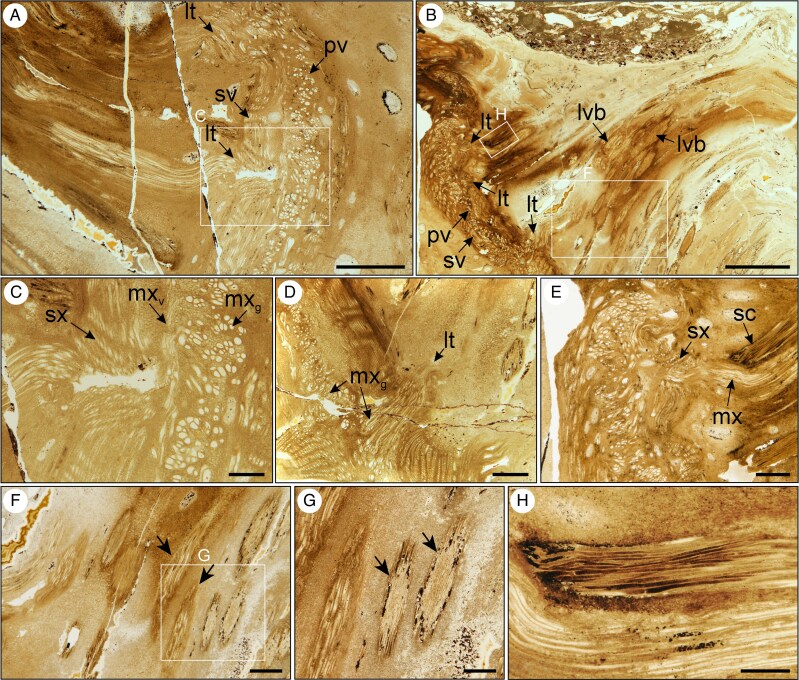
Further anatomical details of M 61c on the stem and frond vasculature. (A) Overview of the active frond petiole attached to the stem on the left side of the section with emerging leaf traces (lt), the primary girdling vasculature with metaxylem clusters cut in cross-section (pv) and the initiation of the secondary vasculature with vertical metaxylem clusters and secondary xylem and phloem (sv). Scale bar: 2 mm. (B) Active frond petiole on the right side of the section, with the peripheral vascular segment (PVS) and emerging leaf traces, and various leaf vascular bundles (lvb) cut in sub-cross-section. Scale bar: 5 mm. (C) Detail of the leaf trace from A, showing the primary girdling metaxylem (mx_g_), the secondarily formed vertical metaxylem (mx_v_) and the secondary xylem of the PVS. Scale bar: 0.5 mm. (D) Detail of leaf trace from B, showing the connection of leaf traces with the primary girdling vasculature. Scale bar: 1 mm. (E) Another leaf trace from B, showing secondary xylem connection of the PVS and that of the leaf trace (sc, sclerenchyma strands). Scale bar: 1 mm. (F) Petiole vascular bundles (arrows). Scale bar: 1 mm. (G) Petiole sclerenchyma strands (arrows). Scale bar: 0.5 mm. (H) Detail of associated vascular bundle and sclerenchyma strands. Scale bar: 0.5 mm.

*Stem-apical section (M 61c):* The stem-apical section measures 74 mm in width and 33 mm in height (Fig. 6A). The silicified uppermost stem has been preserved in volcanic tuff, visible at the top of the section as a heterogeneous sediment composed of glass shards, pumice fragments and fine ash matrix. The transition to the fossil plant body is clear and abrupt. The apical stem part represents a flat dome-shaped, cylindrical structure terminating in a nearly horizontal planar surface that is overtopped by several leaf primordia (Fig. 8A, B). Sidewards attached to the stem, two mature and formerly active fronds are cut in longitudinal section, showing the transition of the stem and frond vasculature (Fig. 9A, B). The apical meristematic zone is represented as a small elevation in the central position of the stem (Fig. 8B). The section shows the central zone of parenchyma, which is on average 22 mm wide and contains numerous mucilage cavities and the apical CAS (Fig. 8L–P). On both sides of the central parenchyma, the apical PVS shows the development of primary and secondary (vascular) tissues (Fig. 9A). From the PVS, leaf traces emerge into the cortex from the primary tissues at different levels along the section (Fig. 9A–E). Their trajectory is indicated by the strands of vascular tissues and the sclerenchyma strands that are longitudinally intersected (Fig. 9F–H).

At the nearly horizontal top surface of the stem, a 2.5-mm-wide and 400-µm-high, cone-like elevation is recognized and interpreted to represent the SAM, where cell initials were potentially located (Fig. 8B). However, cellular details of the SAM are incompletely preserved. As a relict, numerous cellular structures are preserved and filled with black content. These cells become larger and more elongated towards the periphery. The mucilage cavities directly below this region do not exceed 100 µm in diameter and are predominantly elongated horizontally. A clear spatial demarcation is present between the apex and the overlaying leaf primordia, but a distinct tissue contrast between the apex and the directly neighbouring leaf primordia is absent. However, a distinction is possible based on the mucilage cavities, which are distinctly larger and spherical to elliptical in the leaf primordia (Fig. 8A, I–K). The parenchymatous tissues near the apex consist of thin-walled, isodiametric cells, measuring ≤100 µm in diameter.

Several leaf primordia were differentiated in M 61c, both longitudinally and transversely, by the distribution, orientation and size of the abundant mucilage cavities. Three initial leaf primordia are directly overlying the apex. They are 5 mm wide on average and appear to be transversely intersected. On the same horizontal level, a few other leaf primordia extend on both sides, measuring ∼13 mm in length and coiling upwards to form a hook-like structure. Occasionally, single clusters of vascular elements occur, measuring ∼70 µm in diameter and meandering through the parenchymatous tissue (Fig. 8J). The leaf primordia exhibit well-matured mucilage cavities that are horizontally elongated (Fig. 8I, K). The leaf primordium closest to the apex shows two size classes of mucilage cavities, with the larger ones concentrated in the lower part and the smaller ones present at the tip of the leaf primordium. The two tallest leaf primordia extend to both sides of the stem (Fig. 6C, numbers 9 and 10). These primordia are ∼20 mm long and possess numerous xylem clusters, not yet arranged in continuous bundles. They both contain only a few mucilage cavities that are highly elongated and measure ≤1 mm. At the top left of the section, two fragments of ground tissue are visible and are interpreted as belonging to the leaf primordium previously number 9 (Fig. 6A).

In the stem, procambial strands corresponding to the vascular bundles become differentiated 1 mm below the apex, at the interface between the apical meristem and the central zone of parenchyma. In the radial section, the bundles are represented by primary xylem strands cut in cross-sections, thus indicating a horizontal orientation (Fig. 8C). The tracheids of the initial bundles are 20–32 µm wide, later extending to ≤60 µm with vascular development (see Fig. 8D–H). The tracheids are arranged in clusters that follow a horizontal row parallel to the general structure of the apex and become more numerous towards the stem periphery. At the stem periphery, vertically oriented vascular bundles are additionally formed and cut in longitudinal section. At this point, only metaxylem tracheids are visible (Fig. 8H). They measure 50–140 µm and exhibit scalariform to multiseriate araucarian pitting, with the pits being ∼7 µm wide. Further downwards, the vertical metaxylem tracheids are in direct contact with the secondary xylem, at the periphery of the primary vascular area of the PVS. In the centre of the PVS, horizontal metaxylem cells are 60–110 µm wide and grouped in clusters of two to five cells (Fig. 9A). At the interface between parenchyma and primary xylem, elongated mucilage cavities ≤600 µm in width are formed. Elongated mucilage cavities are also visible sporadically inside of the primary vascular network of vertical and horizontal metaxylem cells of the PVS, but their size does not exceed 400 µm there.

The secondary tissues of the PVS start developing 1–2 mm above the uppermost leaf trace and are produced by a bifacial cambium at both sides of the primary vasculature (Fig. 9C, D). The tracheids run predominantly vertically (Fig. 9B), but at the level of leaf traces their orientation is widely horizontal (Fig. 9C–E). The cells are 50–70 µm wide and bear multiseriate araucarian pitting. The secondary phloem is represented by a thin layer of one to two sub-angular sclerenchymatic cells.

The petiolar bases of two attached fronds show the direct connection of the leaf vasculature with the stem vascular system (PVS), in longitudinal section (Figs 6A and 9A, B). Each frond is connected to the stem vasculature by multiple leaf traces that originate from the PVS. Each leaf trace originates from clusters of mainly radially and horizontally oriented metaxylem tracheids leaving the primary tissue area of the PVS towards the cortex (Fig. 9C–E). Each leaf bundle developed secondary xylem and phloem from an individual lateral meristem distal to its connection with the PVS (Fig. 9F). Close to the PVS, the bundles are 1 mm wide but become thinner and exhibit fewer secondary tissues the more distal they are from the PVS. About 10 mm distant from the PVS, the bundles are composed only of metaxylem clusters (Fig. 9F). Here, the vascular bundles are spatially reorganized and grouped in specific areas of the petiole, separated by several parallel sclerenchyma strands (Fig. 9G, H). The sclerenchyma cells of the strands are <1.5 mm long and 35–55 µm wide, with 8- to 12-µm-thick walls, and sporadically contain black components.

A few millimetres below the apex, additional primary xylem clusters are grouped together to form four initial CAS (Fig. 8L–P). A total of 12 longitudinally elongated CAS was counted on the whole section, each measuring 1–2 mm in width and consisting of primary and secondary vascular tissues, away from the apical meristem. The bundles are presumably formed from isolated procambial regions in the pith that are not related to the procambium of the PVS. Close to the apex, these procambial regions form clusters of metaxylem tracheids running in undefined orientations (Fig. 8M, N). The tracheids measure 50–70 µm in diameter and exhibit scalariform to multiseriate araucarian pitting (Fig. 8O). Secondary tissues developed 2 mm below the metaxylem cluster nearest to the apex, co-occurring with predominantly vertically orientated metaxylem clusters (Fig. 8P). The secondary tissues are produced by a bifacial cambium, which developed rows of secondary xylem tracheids alternating with parenchymatous medullary rays. The tracheids are 60–90 µm wide and bear multiseriate-bordered, araucarian-type pitting. The secondary phloem is poorly preserved, with only remnants of sub-angular sclerenchyma cells remaining. Mucilage cavities ≤400 µm in width are present within the medullary rays of the most developed CAS.

Globular to elongate mucilage cavities frequently occur in all investigated sections and are present in the ground tissues of the pith, the cortex, leaf primordia and the mature petioles, but also occur interspersed with primary vascular tissues of the stem (Fig. 5). The orientation of their elongation was probably controlled by growth pressure resulting from directed cell division and an increase in the volume of the tissue. Size differences of the cavities mirror their ontogenetic development. Cavities of elongated shape ≤600 µm wide had already formed directly underneath the apex and above the procambial zone (Fig. 8A). They appear sporadically within the primary vascular tissue area of the PVS, where they do not exceed 400 µm in width (Fig. 9A–E). Within the central zone of parenchyma, the mucilage cavities are almost perfectly spherical and measure 100–1500 µm in diameter (Figs 8L–N, P and 7). Mucilage cavities closely associated with the primary xylem of the CAS are 200 µm wide, on average. In the petiolar parenchymatous ground tissue, mucilage cavities tend to be rare, but are extremely elongated, measuring ∼1 mm in length (Fig. 9A, B).

## Remarks

Weber and Sterzel (1896) defined *M. stellata* var. *corticata* as a taxonomic variety of *M. stellata* stems that are characterized by thin layers of secondary xylem in the PVS and a thick cortex encompassing many vascular bundles, sclerenchyma strands and mucilage cavities. They identified M 61c and M 88a–c as *M. stellata* var. *corticata*, because their PVS possesses only 1- to 2-mm-thick secondary xylem layers and the cortex makes up half of the total stem diameter. In M 88a–c, the secondary xylem zones of the PVS are 1 mm thicker than in M 61c, and the cortex extends only over one-third of the whole stem. As argued above, both specimens of M 61c and K2986 (with M 88a–c) are supposed to be part of the same individual stem, whereby the comparative characteristics of both sections clearly show an ontogenetic trend of more secondary xylem and less cortex tissue towards the stem base. Although the presence of spherical mucilage cavities is typical of all medullosans, their high abundance in both investigated ‘var. *corticata*’ specimens is uncommon, compared with other *M. stellata* var. *typica* stems. Moreover, *M. stellata* var. *typica* stems usually have much larger stem diameters than *M. stellata* var. *corticata* specimens and exhibit thicker layers of secondary xylem. In summary, all these characteristics clearly indicate that *M. stellata* var. *corticata* represents an early ontogenetic stage of *M. stellata* var. *typica*, as already suggested by Weber and Sterzel (1896) in their diagnosis of the *corticata* variety.

From a nomenclatural point of view, we conclude that the use of taxonomic varieties for different ontogenetic stages, as applied by Weber and Sterzel (1896), is invalid. Therefore, we suggest that only the valid taxon name of *Medullosa stellata*Cotta, 1832*emend.*Luthardt *et al.*, 2021 var. *typica*Weber & Sterzel, 1896 should be used for all medullosan stems sharing the corresponding diagnostic features (see Luthardt *et al*., 2021, 2022). The purely descriptive terms of ‘var. *typica*’ and ‘var. *corticata*’ should hereafter be regarded as synonyms. Other varieties, such as *M. stellata* var. *gigantea*, *M. stellata* var. *lignosa* and *M. stellata* var. *incrassate*, require more detailed investigations to define their taxonomic significance.

## DISCUSSION

### Apical meristem structure

**Fig. 10.**
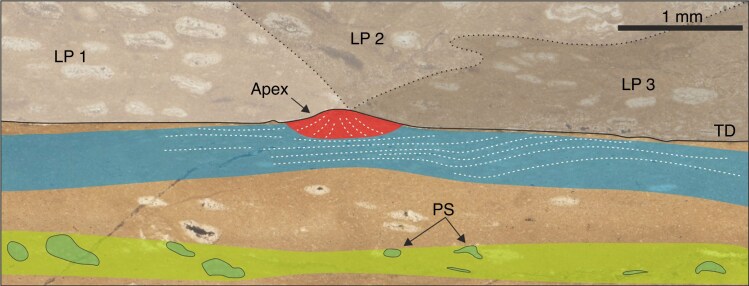
Interpretation of near-apical structures including the apical meristem (red region) with predominantly anticlinal cell division (white-dashed lines), covered by various leaf primordia (LP 1–3) and separated from those by a tissue demarcation line (TD). Blue region indicates the supposed position of a primary thickening meristem based on the horizontal orientation of cells (white-dashed lines), implicating periclinal tissue formation. Greenish zone indicates the position of the provascular cambium and initial provascular strands (PS). Light-coloured spheres in the tissue represent mucilage cavities.

Owing to the exceptionally flat morphology of the K2986 apex, the zone of apical initials is hard to identify. We interpret a 1.5-mm-wide elevation in stem central position as the SAM because it exhibits a clear demarcation against the overlying leaf primordia (Fig. 8B). The diffuse cellular structures with dark fillings show an oblique periclinal growth orientation, and overall size increases away from the tip of the apical dome (Figs 8B and 10). Both the orientation and the increase in size of the cellular structures could point to a zone of multiple actively dividing cell initials, which is more likely than a single apical cell typically occurring in most seed-free plants, e.g. the Polypodiopsida and Lycopsida (Newman, 1965; Good, 1971; Stevenson, 1976; Arnoux-Courseaux and Coudert, 2024). A multizonal apex with a central zone of multiple actively dividing cell initials and an outer initial layer of protodermal cell initials is present in extant seed plants and referred to the simplex type (e.g. Kean-Galeno *et al*., 2024). This specific cellular organization is not confirmed for the apex investigated here, because an outer protodermal zone could not be identified. In conclusion, the apex of *M. stellata* seems to differ from any type of apex in extant plants, by most probably possessing a zone of multiple dividing cells without a protodermal zone. Although the tissue preservation is limited, we assume an unknown, more phylogenetically basal apex type occurring only among ancient spermatophytes. This scenario appears likely when considering that the stele of *M. stellata* shows some major differences compared with the modern eustele of extant spermatophytes (see the below section ‘Evolutionary considerations on medullosan vascular architecture’).

### A primary thickening meristem?

With its nearly horizontal upper demarcation, the apex of K2986 (M 61c) exhibits a remarkable morphology that lacks comparable examples from the extant flora (Figs 6 and 8A). With its initially wide pith and wide stem diameter, it is best compared with apices of extant cycads and, in a wider sense, with those of some extant monocots (Arecaceae). Both extant clades possess a so-called primary thickening meristem (PTM; Stevenson, 1980; DeMason, 1983; Rudall, 1991). A PTM is defined by a diffuse primary meristematic zone that decreases in cross-sectional extent in the basipetal direction and is associated with extensive anticlinal cell files and cell initials predominantly dividing in periclinal planes (DeMason, 1983). In the Arecaceae, the shoot apex itself might be sunken and overarched by the leaf primordia (Rauh and Rappert, 1954; DeMason, 1983). For K2986, we cannot provide direct evidence of a PTM because a zone of anticlinal cell division is not confirmed owing to preservation bias of the parenchymatous tissue. However, the anticlinal orientation of the procambial zone in K2986 is demonstrated by the nearly horizontal files of primary xylem forming the initial stem vascular system (Fig. 8C). A periclinal cell division is also suggested by the predominantly anticlinal elongation of the diffuse cellular structures and mucilage cavities directly below the apex that most probably results from directed stress caused by the increase in volume during tissue formation (Fig. 10). Therefore, the presence of a nearly horizontal PTM in *M. stellata* is probable, with a position between the apical cell initials and the procambial zone (Fig. 10). A PTM leads to important radial growth that results in the formation of pachycaulous stems, as reported for the cycads (Stevenson, 1980) and medullosans (Luthardt *et al.*, 2021). Arborescent plants with a PTM generally exhibit a single unbranched stem and helically attached, frond-like leaves, broadly consistent with Corner’s model of tree architecture (Hallé *et al.*, 1978; DeMason, 1983). The same architectural model was suggested for the *M. stellata* plant, based on another stem apex with attached fronds from the same locality (Luthardt *et al.*, 2022). Moreover, reproductive organs in connection with Permian medullosan stems are unknown (Luthardt *et al.*, 2021), and no evidence of growth interruption was found in the fossil record, thus removing Chamberlain’s model from the most probable architectural models attributable to *Medullosa*. Primary stem thickening might result in reduced vertical growth rates of the stem. A direct comparison of PTM with normal SAM growth rates was made in fast-growing long shoots (normal SAM) and slow-growing short shoots (PTM) of conifers (Gifford and Wetmore, 1957). Slow stem heightening is also observed in arborescent cycads (Whitelock, 2002).

### Leaf arrangement at the apical meristem

Various leaf primordia of different ontogenetic stages are arranged in a nearly horizontal succession, thus forming a protective parenchymatous layer completely covering the apex (Figs 6A, C and 8A). In this layer, a clear differentiation of single primordia is challenging because clear demarcations are mostly missing. Again, the structural arrangement and orientation of mucilage cavities are helpful in defining the separate organs, supported by occasionally occurring provascular elements of the primordia. At least two of the primordia have a hook-shaped morphology in longitudinal section, which points to a coiled habit. Coiled leaf primordia result from a circinate vernation, which occurs widely in ancient and extant ferns (e.g. Cruz and Hetherington, 2025), but also in the leaves and leaflets of some extant cycads (Chamberlain, 1919; Stevenson, 1981). In cycads, the circinate vernation is assumed to represent a synapomorphy among some clades (Griffith *et al.*, 2014). For medullosans, only sparse information exists on the early ontogeny of their extensive fronds. Circinate and conduplicate vernation has been described for some foliage taxa of the Carboniferous assigned to the medullosans and lyginopterids (e.g. Cleal and Laveine, 1988; Krings *et al.*, 2008). The specimen investigated here demonstrates that circinate vernation persisted as a general feature in medullosans even in the arborescent taxa of the early Permian. This feature might be interpreted as a plesiomorphic character of seed-ferns, as for the cycads, or simply as a homoplasy in the development of large, compound leaves.

### Vascular architecture of the stem apex

The position of the procambium is demonstrated indirectly by the presence of primary vascular elements, initially formed ∼1 mm below the supposed apex (Fig. 10). The initial stem vasculature comprises various isolated clusters of low-diameter primary xylem tracheids in the central position, which develop centrifugally into wider metaxylem tracheids (Fig. 8D–H). The clusters have irregularly arranged tracheids and no phloem, thus differing greatly from collateral bundles in ‘modern’ eustelic stems, such as those commonly found in extant gymnosperms and angiosperms (Devadas and Beck, 1972). In the section, the clusters are arranged initially in a horizontally oriented vascular system that changes its orientation distinctly into a vertical orientation close to the stem periphery (Figs 6B and 9A).

**Fig. 11.**
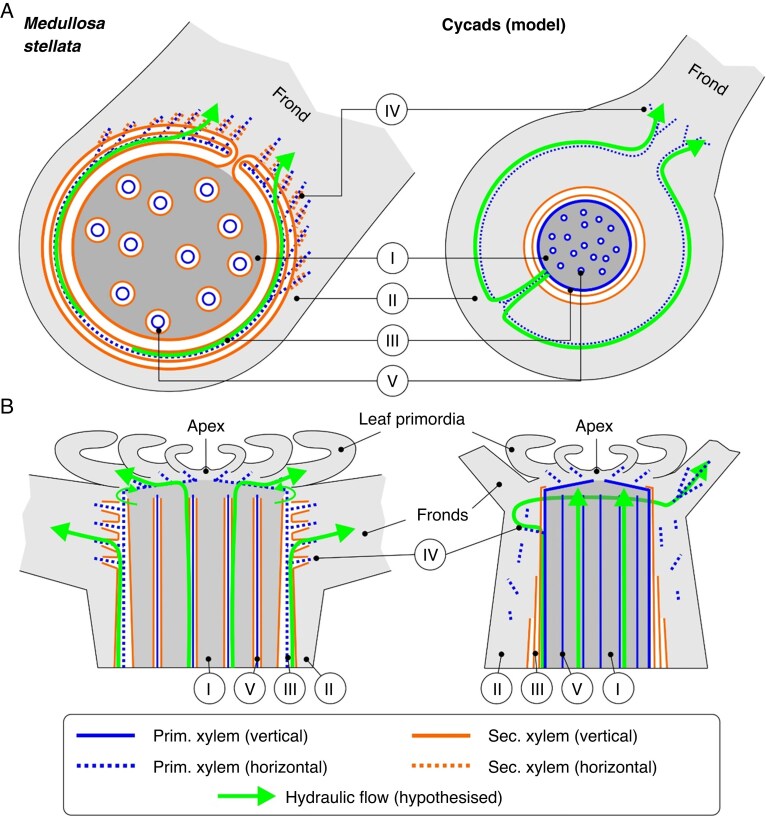
Comparison of the stem vasculature of *Medullosa stellata* with that of a model cycad (comparable to *Encephalartos altensteinii*) to demonstrate functional similarities of the hydraulic system and the hypothesized directions of hydraulic flow, in cross-section (A) and radial view (B). Abbreviations: I, wide parenchymatous pith; II, thick cortex; III, peripheral vascular system, from which leaf traces are produced; IV, leaf traces or girdling traces in cycads; V, pith strands, or central accessory strands (CAS) in *Medullosa* and medullary bundles in cycads.

The horizontal orientation of the initial vasculature is an uncommon feature occuring in *M. stellata*. In parallel, the horizontal orientation of the tracheids in the initial PVS is striking. The metaxylem tracheids presumably follow a horizontal course, tangentially oriented in a basipetally widening stem vasculature, thus encircling the whole stem in its circumference (see Figs 4D and 5D, E). This ‘girdling vasculature’ is also present in the PVS of nearly all cross-sections of adult medullosan stems, including all varieties of *M. stellata*, *M. leuckarti* and *M. porosa* (Rudolph, 1922; Luthardt *et al.*, 2021). The sections show that the leaf traces emerge from the tangential and horizontal primary tissues of the PVS encircling the stem, thus demonstrating the direct connection of the PVS with the frond vasculature (Figs 4D, 5E, 9D and 11). The connection already existed between the leaf primordia and the stem vasculature in the procambial phase (Fig. 8G). Vertically oriented vascular elements, including metaxylem bundles and secondary xylem strands, were formed at a later ontogenetic stage of the PVS, when the leaf primordia became mature fronds. In comparison, procambial strands in Eudicots are formed vertically, directly under the shoot apical meristem (Claßen-Bockhoff *et al.*, 2021).

### The hydraulic system of medullosans

The sections investigated here provide exclusive insights into the development of the complex and uncommon vascular system of early Permian medullosans. Its hydraulic functioning and evolutionary significance have remained widely enigmatic since their first description by Cotta (1832). In particular, the co-occurrence of a PVS with its horizontal metaxylem tracheids (primary ‘girdling’ vasculature) and the CAS have been subject to discussion (e.g. Rudolph, 1922). From the new data of K2986, we present here a first comprehensive hypothesis on the functioning of the hydraulic system in *M. stellata* and other related taxa.

So far, we could demonstrate that the leaf primordia were connected directly with the initial horizontal procambial network of the PVS. Moreover, the leaf traces of adult fronds are also predominantly formed by the ‘girdling vasculature’. As pointed out by Rudolph (1922), a horizontal conducting network is inefficient in transporting water vertically from the roots into the leaves. M 61c shows that the PVS formed a vertical vasculature at the attachment position of the petiole, like that of gymnosperms and woody dicots (Lucas *et al.*, 2013), but comprising metaxylem clusters and secondary xylem with phloem from bifacial cambia, to both sides of the PVS.

The secondary vasculature contributed significantly to the water supply of the numerous leaf traces forming the large compound leaves (fronds that could reach 5 m in length) of medullosans, in two ways (Fig. 11): (1) a direct network connection with the girdling metaxylem tracheids of the primary vasculature in the primary tissue zone of the PVS (see also Rudolph, 1922); and (2) the direct connection of the PVS secondary xylem with the secondary xylem of the initially emerging leaf traces. In summary, the adult fronds of *M. stellata* were predominantly supplied hydraulically by the primary and secondary vasculature of the widely developed PVS, similar to the omega system of cycads, with the girdling leaf traces emerging from the primary tissues of the stem (Tomlinson *et al.*, 2018).

In contrast to the well-developed vasculature of the PVS, the near-apical procambial vasculature lacks any tracheid that would be capable of efficient vertical water transport to the developing leaf primordia. However, a few millimetres below the provascular zone the CAS are initiating by widely chaotically arranged primary vascular bundles that rapidly turn into a predominant vertical orientation, downwards. The CAS formed a network of stelar strands that developed secondary xylem and phloem, in addition to their primary xylem. The connection of this strand network with the overall stem-conductive network remained unknown, so far, although the CAS certainly contributed to the water-conducting system of *M. stellata* (see Luthardt *et al.*, 2021, 2022). However, the apical section of M 61c does not show the CAS directly connected with the frond-feeding vasculature of the PVS. Instead, we assume here that the CAS were transporting water to the parenchymatous apex and were thus supporting the initial ‘girdling vasculature’ of the leaf primordia by short-distance diffusion through the parenchyma cells (Fig. 11B).

In summary, the peripheral vascular system of *M. stellata* was supplied by the CAS at an early ontogenetic stage of leaf primordia, whereas the secondary vasculature of the PVS was mainly supplying the fronds at mature stage. This new hydraulic hypothesis for *M. stellata* draws the picture of a highly complex and evolutionarily unique mode of vascular supply that is not comparable to any kind of vascular system among extant spermatophytes (Decombeix *et al.*, 2019), with the sole exception of the Cycadales.

### Evolutionary considerations on medullosan vascular architecture

The new insights into the vascular development in an apex of the Permian spermatophyte *M. stellata* shed light on the stelar evolution of seed plants, in relationship to extant clades. Among the latter, the Cycadales have been compared repeatedly with the Medullosales, back to historical investigation periods (e.g. Worsdell, 1906; Scott, 1909; Bancroft, 1914; Rudolph, 1922; Luthardt *et al.*, 2021). The wide anatomical similarities of early Permian medullosans to cycads gave reason to classify them as sister groups, for some authors (Göppert and Stenzel, 1881; Solms-Laubach, 1897). In fact, the whole stem vascular system of *M. stellata* and that of various cycads show similarities in complex traits, including their ontogeny (Fig. 11). These generally comprise the combination of: (1) a flat apex developing from a PTM and forming a pachycaulous stem that possesses a wide pith and large amounts of parenchyma, and conforming to the architectural corner model, which is also true for various cycads (Hallé *et al.*, 1978); (2) a complex primary vasculature including tangentially and horizontally oriented conducting elements; (3) additional vascular support by vascular bundles embedded in the pith; (4) manoxylic secondary xylem with continuous or medullary rays and araucarian-type pitting; and (5) the frequent occurrence of shizo-lysogenous mucilage cavities or canals (see the below section ‘Significance of the mucilage cavities’).

Given that the arborescent growth is evidenced for the Chemnitz medullosans (Luthardt *et al.*, 2021, 2022), we propose that the primary similarity of *M. stellata* and cycads was the overall growth model of a pachycaulous stem to realize arborescent growth without considerable secondary thickening as occurring in most woody seed plants. In contrast, early medullosans of the Carboniferous had slender stems, with few isolated vascular bundles (e.g. Basinger *et al.*, 1974; Dunn, 2006; Luthardt *et al.*, 2021), growing as small self-supporting plants or as vines with elongated stems (e.g. Dunn *et al.*, 2003; Krings *et al.*, 2003), thus most probably lacking a primary stem thickening. To grow as taller trees in seasonally dry forests of the early Permian, medullosans required distinctly wider stems that were primarily developed by a putative PTM, in the case of *M. stellata*. The stem primary thickening probably played a key role in providing the balance between structural stability and effective water conductance and circulation through the stem, comparable to what can be achieved by extant arboreal taxa such as gymnosperms and woody dicots (Lucas *et al.*, 2013). But in *M. stellata*, this balance was achieved, in part, by the structural support of the overall cylindrical secondary growth, initiated by the early development of the PVS from the PTM. Consequently, we can assume that primary stem thickening was not an apomorphic feature of all medullosans but an evolutionary innovation within the clade that first occurred in arborescent taxa of the early Permian. Although the sections investigated here provide the oldest indication of a PTM in spermatophytes, we cannot exclude the possibility that early cycads of the same age and similar habitats also had a PTM, because their anatomy was broadly similar to that of extant cycads (Spiekermann *et al.*, 2021; Luthardt *et al.*, 2023). As a developmental consequence, a primary thickening stem produces leaf primordia that are arranged around the apical meristem on its quasi-horizontal flanks (Fig. 11B). In adaptation to this configuration, a horizontal water-conducting system was necessary to supply the primordia, because of a cellular interface separating the leaf primordia from the secondary vertical vascular system located below (Fig. 8A). In cycads, horizontal transport is realized by the girdling leaf traces, whereas in medullosans the primary encircling vasculature of the PVS was responsible for horizontal water transportation (Fig. 11A). The wide parenchymatous apex and the horizontal girdling vasculature also need additional vertical vascular support for efficient transfer of nutrients from the roots to the apex, which is implemented by the CAS in medullosans and by the medullary bundles in various cycads (Fig. 11B) (Worsdell, 1901; Chamberlain, 1911; Greguss, 1968).

As a general conclusion, most of the specific anatomical similarities of cycads and *M. stellata* might be regarded as functional analogies. As a result, different anatomical units have had the same hydraulic functions, e.g. cycad girdling leaf traces and the medullosan encircling vasculature of the PVS. This hypothesis is also supported by the fact that early medullosans exclusively possessed a vertical vasculature without horizontal conducting elements and CAS (e.g. Dunn, 2006), reflecting an evolutionary development within the clade of Medullosales. Another major difference might be seen in the overall stele characteristics. The Cycadales exhibit a eustele typical of extant seed plants, with a regular leaf trace production and collateral bundles (Greguss, 1968). For the Medullosales, the stele type has been a matter of discussion. At the beginning of the 20th century, it was thought that *Medullosa* had a polystelic solenostele or a complex dictyostele (Seward, 1917), as in the Polypodiopsida. But based on the regular formation of leaf traces, Basinger *et al.* (1974) suggested that *Medullosa* had a monostele, suggesting a close relationship with gymnosperm eusteles. Although we agree with the monostelic interpretation, we need to emphasize the differences between a typical eustele as occurring in cycads and the *M. stellata* stele; these are as follows: (1) the absence of collateral bundles in the stem (instead, chaotically organized metaxylem clusters without phloem); (2) the specific tissue arrangement of the PVS, with secondary xylem and phloem growing centrifugally and centripetally from the primary vasculature; (3) the production of numerous leaf traces supplying one compound leaf; and (4) a distinct gap in the PVS always associated with an attached petiole base. We interpret most of these divergences as ancestral features rather typical of an amphiploic siphonostele, including the secondary tissue arrangement of the PVS, dispersed metaxylem clusters, and a leaf gap-like interruption in the PVS. Therefore, the *Medullosa* stele most probably represents an ancestral type of monostele that shares structural features of both siphonosteles and eusteles (Tomescu, 2021).

### Significance of the mucilage cavities

Spherical to elongate mucilage cavities frequently occur in the stem-apical sections of *M. stellata*, with a wide variety in shape and size. The cavities represent empty spaces created by the surrounding parenchyma cells of the ground tissue that were filled with water or secretions (Fig. 7). In general, two types are described for the formation of mucilage cavities: the schizogenous type and the lysigenous type. Schizogenous cavities are formed by the consecutive separation of the common parenchyma walls, whereas lysigenous cavities are formed by destruction of parenchyma cell walls, leaving a free space into which the cytoplasm is discharged and changes its composition to become mucilage (Joel and Fahn, 1980). An intermediate type occurs in cycads, where mucilage cavities are schizo-lysigenous, meaning that the common wall of neighbouring parenchyma cells is separated, before it is destroyed (Greguss, 1968). In *Medullosa*, the cellular structures point to the same intermediate type, with broken cell walls occurring among the crushed cells at the periphery of the cavities. However, further details are lacking to determine whether the common cell walls separated to create the cavity.

Mucilage cavities generally occur in all medullosan stems (e.g. Scott, 1899; Steidtmann, 1944; Hamer and Rothwell, 1988) and in the closely related genera *Sutcliffia* Scott, *Quaestora* Mapes et. Rothwell, and *Colpoxylon* Brongniart (Grambast, 1962). In most of these, mucilage cavities have been described only in the cortex, where they were associated with sclerenchyma strands in the petiolar bases. Only in *Sutcliffia*, mucilage cavities were also observed in the primary vascular tissue and in the parenchymatous rays (Scott, 1906; De Fraine, 1912). Additionally, numerous spherical mucilage cavities were described from various regions of the stem apex of *Callistophyton poroxyloides* (Delevoryas, 1956). In general, the occurrence of spherical mucilage cavities might be a specific feature of medullosans and related clades or taxa.

For extant Cycadales, Greguss (1968) described in detail the mucilage cavities, which are found in the pith, the broader primary pith rays, the cortex and the leaf vasculature and lamina (see also Coiro *et al.*, 2020). Fossil cycads from the Mesozoic exhibit the same distribution of mucilage cavities in the stem and fronds (Smoot *et al.*, 1985; Martínez *et al.*, 2012).

Various types of secretory cavities and canals are present in many tracheophytes, including Polypodiopsida (Brebner, 1895), Lycopodiopsida (Bruce, 1976), angiosperms (Classen *et al.*, 1998) and gymnosperms (Mastroberti and De Araujo Mariath, 2008). Their function varies depending on their location inside the plants and is also influenced by the external climatic conditions. In stipules of angiosperms, mucilage cavities function as nutrient storage structures (Horner and Lersten, 1968). In roots, mucilage forms a protective layer against external threats found in the soil, such as fungi or bacteria (Rougier and Chaboud, 1985). In the needle-bearing shoots of conifers growing in climates with seasonal variation, the mucilage cavities are used to store water and help with the regulation of water transport and irrigation of the needles to protect against frost (Distelbarth and Kull, 1985). In cycads, they contribute to the water storage and provide protection against external threat in case of injury (Greguss, 1968). In addition, they produce neurotoxins that act as defence compounds against herbivores (Brenner *et al.*, 2003). Their role in *M. stellata* remains speculative but might have been similar to that in cycads, because they are present mostly near the apex and all around the crown of active fronds. This sensitive region of primary growth continuously requires sufficient nutrients and water; even more so, given that the medullosans of Chemnitz were thriving in seasonally dry conditions (Luthardt *et al.*, 2016, 2021). In addition, mucilage cavities were probably storing specific substances to protect the apex and leaf primordia against herbivory and potential injuries, which is suggested by their high concentrations in these tissue regions.

## Conclusion

The assessment of primary growth processes in an early Permian pteridosperm provides initial insights to ancient modes of apical meristematic activity and the early vascular development in basal seed plants. The stem apex of *M. stellata* exhibits various exceptional features that are mostly referred to its basal position within the seed plant clade, including: (1) a putative unusual arrangement of cell initials in the apical meristem; (2) a complex vasculature, with a primary girdling and a secondary vertical conducting network; and (3) an unusual type of a monostele that exhibits features of both siphonosteles and eusteles. Consequently, the *Medullosa* stele investigated here might demonstrate that the stelar evolution of Palaeozoic spermatophytes was a non-continuous process, generating various hybrid forms of stele types, in adaptation to various growth models. Among them, primary stem thickening is a specific strategy of arborescent growth that evolved independently in extant and extinct seed plant clades, but also in the Monilophytes and extinct Lycopsida. The complex vascular system of *M. stellata* results from the primary stem thickening and the corresponding position of the leaf primordia. The comparison with extant cycads demonstrates that similar stem anatomical features of both clades are likely to be referred to functional analogies of the vascular systems that evolved independently in response to the specific growth architecture that includes a pachycaulous stem. Nevertheless, other shared features of early Permian medullosans and cycads, such as the manoxylic wood sometimes developing into several polyxylic segments, the high amount of specific mucilage cavities distributed throughout the whole stem or the presence of vascular bundles in the pith, indicate a close phylogenetic relationship, with the medullosans representing the more ancestral clade.
